# Predicting lymph node metastasis using preoperative parameters in patients with T1 ampulla of vater cancer

**DOI:** 10.1186/s12885-024-12311-9

**Published:** 2024-08-01

**Authors:** So Jeong Yoon, Seung Soo Hong, Kee-Taek Jang, So Kyung Yoon, Hongbeom Kim, Sang Hyun Shin, Jin Seok Heo, Chang Moo Kang, Kyung Sik Kim, Ho Kyoung Hwang, In Woong Han

**Affiliations:** 1 Division of Hepatobiliary-Pancreatic Surgery, Department of Surgery, Samsung Medical Center, Sungkyunkwan University School of Medicine, 81 Irwon-ro, Gangnam-gu, Seoul, Korea; 2 Division of Hepatobiliary and Pancreatic Surgery, Department of Surgery, Severance Hospital, Yonsei University College of Medicine, 50-1 Yonsei-ro, Seodaemun-gu, Seoul, Korea; 3 Department of Pathology and Translational Genomics, Samsung Medical Center, Sungkyunkwan University School of Medicine, Seoul, Korea; 4 Department of Surgery, Soonchunhyang University Seoul Hospital, Soonchunhyang University College of Medicine, Seoul, Korea

**Keywords:** Ampulla of vater cancer, Ampullary cancer, Lymph node metastasis, Prognosis

## Abstract

**Background:**

Lymph node (LN) metastasis is an established prognostic factor for patients with surgically resected ampulla of Vater (AoV) cancer. The standard procedure for radical resection, including removal of regional LNs, is pancreaticoduodenectomy (PD); however, local excision has been considered as an alternative option for patients in the early stage cancer with significant comorbidities. In the present study, we elucidated the preoperative factors associated with LN metastasis to determine the appropriate surgical extent for T1 AoV cancer.

**Methods:**

We included patients who underwent surgery for T1 AoV cancer at Samsung Medical Center and Severance Hospital between 2000 and 2019. Risk factors were analyzed to identify the preoperative parameters associated with LN metastasis or regional LN recurrence during follow-up. Finally, using the identified risk factors, a prediction model was constructed.

**Results:**

Among 342 patients, 311 patients underwent PD, whereas 31 patients underwent transduodenal ampullectomy. Fourty-eight patients had LN metastasis according to pathology report, and two patients presented with regional LN recurrence. Age, carbohydrate antigen 19 − 9 (CA 19 − 9), and tumor differentiation were identified as factors associated with the increased risk of LN metastasis or regional LN recurrence. The area under the curve of the prediction model with these three factors was 0.728.

**Conclusion:**

Our newly developed prediction model using age, CA 19 − 9, and tumor differentiation can help select patients who require PD over local excision. Nevertheless, additional in-depth analysis is warranted to select appropriate surgical extent for patients with presumed T1 AoV cancer.

## Background

As a rare malignant condition, ampulla of Vater (AoV) cancer accounts for 0.2% of all gastrointestinal tumors [1]. The only curative treatment is complete surgical resection with negative margins. Tumor histology, lymph node (LN) metastasis, and tumor markers such as carbohydrate antigen 19 − 9 (CA 19 − 9) were reported as prognostic factors of surgically resected AoV cancer [2–4].

Pancreaticoduodenectomy (PD) with regional LN dissection is the standard surgical modality. Although there have been advances in surgical techniques and perioperative management, PD is associated with high morbidity and mortality, with reported morbidity and mortality rates of approximately 40% and 2%, respectively, in recent studies [5, 6]. Considering that patients in their 60s and 70s are frequently affected by AoV cancer [7], transduodenal ampullectomy (TDA) has been considered as an surgical option for high-risk patients with early-stage cancer [8, 9]. However, information on the selection criteria for TDA and its oncological safety regarding the possibility of LN metastasis remains considerably limited.

In this study, we (1) investigated the factors associated with the high probability of LN metastasis or regional LN recurrence in patients with surgically resected early-stage AoV cancer and (2) developed a prediction nomogram to assess the potential of LN metastasis to support decision-making on the appropriate surgical extent of early AoV cancer.

## Materials and methods

### Patient database

We enrolled patients who underwent surgical resection for AoV cancer at Samsung Medical Center and Severance Hospital between January 2000 and December 2019. In total, 342 patients who were pathologically diagnosed with T1 (Tis, T1a, or T1b) stage disease were included.

We retrospectively reviewed the demographic and perioperative clinical characteristics. Laboratory data, including CA 19 − 9, serum amylase, serum lipase, and serum total bilirubin, were collected. The inflammatory markers, neutrophil-to-lymphocyte ratio (NLR) and platelet-to-lymphocyte ratio (PLR) were calculated. Preoperative esophagogastroduodenoscopy, computed tomography, and magnetic resonance imaging were performed to evaluate resectability. Furthermore, the results of preoperative endoscopic biopsy were collected.

All surgical specimens during surgery of the patients were reviewed by the pathologists who took charge of hepatobiliary diseases in each institution. The final pathology report included tumor stage based on the recent American Joint Committee on Cancer (AJCC) system [10], LN status, and tumor differentiation (well-differentiated, moderately differentiated, and poorly differentiated). In terms of tumor differentiation, the final grade was decided by the predominant component if there existed more than two morphological grades.

### Surgical management

The surgeons and anesthesiologists assessed the operative risk in all patients. Perioperative cardiovascular risk evaluation was done according to the American Heart Association (AHA) guideline [11]. The ARISCAT risk index was used to assess the risk of postoperative pulmonary complications [12]. TDA was considered as the first option in the following cases; high-risk patients (> 20%) with perioperative cardiovascular or pulmonary morbidities, or patients with suspected early AoV cancer presenting no suspicious LNs in preoperative imaging studies.

In TDA, a lateral duodenotomy incision was made to identify the tumor of AoV. After resection of the ampullary lesion, the reconstruction of the common channel of common bile duct and pancreatic duct was performed. When the intraoperataive frozen biopsy confirmed tumor-free margin status, the duodenotomy site was closed in a transverse manner.

If the intraoperative frozen section exhibited margin involvement during TDA, PD was performed. By LN dissection during PD, LN stations No. 5 (suprapyloric), 6 (infrapyloric), 8 (common hepatic artery), 12 (bile duct and cystic duct), 13 (posterior aspect of pancreas head), 14 (superior mesenteric artery), and 17 (anterior surface of pancreas head) were removed. During TDA, basically no intentional LN dissection was performed. In some cases, sampling of LNs around hepatoduodenal ligament was done.

### Recurrence and survival

Recurrence was diagnosed based on an increase in CA 19 − 9 levels and the presence of suspicious lesions in follow-up imaging studies, including positron emission tomography. The recurrence site was categorized into two types: loco regional (soft tissue infiltration around the operative bed or regional LNs) and systemic (distant organ metastasis or peritoneal seeding). Regional LNs were the LNs located around the common bile duct, common hepatic artery, portal vein, pylorus, proximal mesenteric arteries, superior mesenteric vein, and right side of the superior mesenteric artery [10].

Recurrence-free survival (RFS) was defined as the time interval between the operation date and the date when recurrent tumors were first identified in imaging studies. Overall survival (OS) was calculated as the time from the operation date to the date of death owing to any reasons.

### Statistical analysis

The Student’s t-test and Chi-squared test were used to compare the clinicopathological variables of the patient groups. The factors associated with RFS were identified using univariable and multivariable Cox regression analysis. Hazard ratios (HRs) were reported with 95% confidence intervals (CIs). The variables associated with LN metastasis or regional LN recurrence were identified using binary logistic regression models with odds ratios (ORs). The factors exhibiting statistical significance with a *p-*value of < 0.05 in the multivariable model were included in a prediction nomogram. The area under the curve (AUC) was used to evaluate the prediction power of the nomogram. All statistical analyses were performed using SAS software, version 9.4 (SAS Institute Inc., Cary, NC, USA.) and R software, version 4.0.5 (R Project for Statistical Computing).

## Results

Among the 342 patients who were included in this study, 311 (90.9%) underwent PD, and 31 (9.1%) patients underwent TDA. The clinicopathological characteristics of the patients who underwent PD and TDA are compared and summarized in Table 1. Preoperative laboratory examinations revealed that the mean values of PLR, serum amylase, and lipase were higher in patients with PD than in those undergoing TDA (157.3 vs. 126.6, *p* = 0.033; 107.3 U/L vs. 71.2 U/L, *p <* 0.001; and 177.9 U/L vs. 56.3 U/L, *p <* 0.001, respectively). Furthermore, more patients with the American Society of Anesthesiology (ASA) score III were present in the TDA group than in the PD group (35.5% vs. 11.6%, *p* = 0.001). Preoperative biopsy was performed in 83.3% and 100% of the patients in the PD and TDA groups, respectively (*p* = 0.007). More patients in the TDA group had well- or moderately differentiated tumors compared with the PD group (67.7% vs. 29.7%, *p <* 0.001). In the final pathology report, the proportion of patients with Tis tumors was higher in the TDA group than in the PD group (35.5% vs. 4.5%, *p <* 0.001). LN dissection was performed in 99.7% of the patients in the PD group and 48.4% of the patients in the TDA group (*p <* 0.001). The major complication rate and length of hospital stay were comparable between the two groups. Recurrence and survival were not significantly different between both groups.

**Table 1:** Comparisons of the clinicopathological characteristics between patients with pancreaticoduodenectomy (PD) and transduodenal ampullectomy (TDA)

Variables	PD group (*n* = 311)	TDA group (*n* = 31)	*p*
*Clinical characteristics*			
Age at operation (year), mean	62.2 (± 9.9)	62.4 (± 11.9)	0.941
Sex, male	160 (51.4%)	16 (51.6%)	0.986
BMI (kg/m2), mean	23.6 (± 3.1)	24.6 (± 3.4)	0.077
Preop. jaundice, yes	85 (27.3%)	6 (19.4)	0.338
Preop. biliary drainage, yes	152 (49.0%)	10 (32.3%)	0.075
Preop. CA 19 − 9 > 37 U/mL	66 (22.0%)	7 (22.6%)	0.941
Preop. NLR, mean	2.3 (± 2.5)	1.8 (± 0.9)	0.197
Preop. PLR, mean	157.3 (± 73.2)	126.6 (± 45.6)	0.033
Preop. serum amylase (U/L), mean	107.3 (± 112.0)	71.2 (± 24.3)	< 0.001
Preop. serum lipase (U/L), mean	177.9 (± 449.8)	56.3 (± 40.7)	< 0.001
Preop. tumor size (cm), mean	1.6 (± 0.8)	1.4 (± 0.6)	0.387
Preop. biopsy, yes	259 (83.3%)	31 (100%)	0.070
Well or Moderately differentiated	77 (29.7%)	21 (67.7%)	< 0.001
Poorly differentiated	169 (65.3%)	10 (32.3%)	
Unknown	13 (5.0%)	0 (0)	
ASA score			0.001
I - II	275 (88.4%)	20 (64.5%)	
III	36 (11.6%)	11 (35.5%)	
*Pathologic characteristics*			
Tumor size (cm), mean	1.6 (± 1.0)	1.5 (± 0.8)	0.678
T stage (AJCC 8th)			< 0.001
Tis	14 (4.5%)	11 (35.5%)	
T1a	224 (72.0%)	14 (45.2%)	
T1b	73 (23.5%)	6 (19.4%)	
LN dissection, yes	310 (99.7%)	15 (48.4%)	< 0.001
The number of harvested LNs, mean	17.8 (± 9.6)	8.9 (± 6.3)	< 0.001
LN metastasis, yes	47 (15.2%)	1 (6.7%)	0.707
Tumor differentiation			0.378
Well or Moderately differentiated	259 (90.9%)	17 (100.0%)	
Poorly or Undifferentiated	26 (9.1%)	0 (0)	
Lymphovascular invasion, yes	65 (30.8%)	1 (7.1%)	0.071
Perineural invasion, yes	15 (7.7%)	2 (15.4%)	0.290
*Operative and Oncologic outcomes*			
Major complications*, yes	69 (22.2%)	4 (12.9%)	0.229
Length of stay (days), mean	16.4 (± 9.5)	13.8 (± 7.6)	0.139
Adjuvant treatment, yes	30 (9.7%)	4 (12.9%)	0.533
Recurrence, yes	64 (20.6%)	7 (22.6%)	0.757
5-year recurrence-free survival rate	78.8%	70.9%	0.594
5-year overall survival rate	80.5%	80.9%	0.995

*PD* pancreaticoduodenectomy, *TDA* transduodenal ampullectomy, *BMI* body mass index, *Preop*. Preoperative, *NLR* neutrophil-to-lymphocyte ratio, *PLR* platelet-to-lymphocyte ratio, *ASA* American Society of Anesthesiology, *AJCC* the American Joint Committee on Cancer, *LN* lymph node

Next, risk factor analysis was performed to identify the factors associated with RFS and OS (Table 2). Multivariable analysis of RFS revealed preoperative serum lipase (HR: 1.002, 95% CI: 1.001–1.003, *p* = 0.003), LN metastasis (HR: 5.879, 95% CI: 2.698–12.812, *p <* 0.001), and tumor differentiation (HR: 3.413, 95% CI: 1.335–8.724, *p* = 0.010) as statistically significant risk factors. Age (HR: 1.037, 95% CI: 1.009–1.066, *p* = 0.009), preoperative NLR (HR: 1.192, 95% CI: 1.092–1.301, *p <* 0.001), and LN metastasis (HR: 2.575, 95% CI: 1.177–5.635, *p* = 0.018) were significantly associated with OS in the multivariable analysis. The patients groups with pathological LN metastasis and regional LN recurrence during follow-up were combined and defined as patients with the potential of LN metastasis (*n* = 53).

**Table 2:** Cox regression analysis for recurrence-free and overall survival in all patients with T1 ampulla of Vater cancer (*n* = 342)

Variables	Recurrence-free survival				Overall survival			
	Uni *p*	HR	95% CI	Multi *p*	Uni *p*	HR	95% CI	Multi *p*
Age at operation	0.859				0.001	1.037	1.009–1.066	0.009
Sex, male (ref. female)	0.616				0.283			
BMI	0.510				0.374			
Preop. jaundice	0.033				0.019			
Preop. CA 19 − 9	0.001				0.007			
Preop. NLR	0.044				0.001	1.192	1.092–1.301	< 0.001
Preop. PLR	0.127				0.207			
Preop. serum amylase	0.001				0.001			
Preop. serum lipase	0.006	1.002	1.001–1.003	0.003	0.046			
Preop. tumor size	0.713				0.913			
Preop. tumor differentiation	0.504				0.861			
ASA score, III (ref. I-II)	0.731				0.062			
Operation, TDA (ref. PD)	0.668				0.995			
Tumor size at pathology	0.575				0.464			
T stage (AJCC 8th)	0.161				0.963			
LN dissection	0.886				0.997			
LN metastasis	< 0.001	5.879	2.698–12.812	< 0.001	< 0.001	2.575	1.177–5.635	0.018
Tumor differentiation	< 0.001	3.413	1.335–8.724	0.010	0.007			
Lymphovascular invasion	< 0.001				< 0.001			
Perineural invasion	0.008				0.252			
Major complication	0.622				0.671			
Adjuvant treatment	< 0.001				< 0.001			

*BMI* body mass index, *Preop*. Preoperative, *NLR* neutrophil-to-lymphocyte ratio, *PLR* platelet-to-lymphocyte ratio, *ASA* American Society of Anesthesiology, *TDA* transduodenal ampullectomy, *PD* pancreaticoduodenectomy, *AJCC* the American Joint Committee on Cancer, *LN* lymph node

Table 3 presents the pathological nodal status and recurrence patterns. Forty-seven patients in the PD group and one in the TDA group presented with pathological LN metastasis. During the follow-up period, 64 (20.6%) patients in the PD group and 7 (22.6%) patients in the TDA group exhibited disease recurrence. Among them, nine patients in the PD group presented with regional LN recurrence.

**Table 3:** The nodal status in final pathology and the patterns of recurrence

Operation	LN status at initial pathology		Recurrence during follow-up	Loco-regional recurrence		Distant metastasis
				Infiltration at operative site	Regional LN	
PD (*n* = 311)	Nx	1	0	0	0	0
	LNM (-)	263	35	5	**5** ^**a**^	25
	**LNM (+)**	**47** ^**a**^	29	3	**4** ^**a**^	22
TDA (*n* = 31)	Nx	16	3	2	0	1
	LNM (-)	14	3	0	0	3
	**LNM (+)**	**1** ^**a)**^	1	0	0	1

*PD* pancreaticoduodenectomy, *TDA* transduodenal ampullectomy, *LN* lymph node, *LNM* lymph node metastasis

^a^Patients with potential of lymph node metastasis (Patients with lymph node metastasis identified at final pathology or with regional lymph node recurrence during postoperative follow-up)

**Fig. 1:**
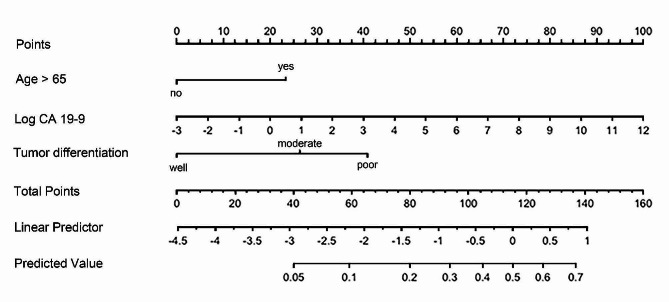
A nomogram calculating the potential of lymph node metastasis using preoperative parameters in patients with T1 ampulla of Vater cancer

Binary logistic regression analysis was performed to investigate the preoperatively measurable factors increasing the potential of LN metastasis (*n* = 53) (Table 4). The cut off value for age at operation with a maximal AUC value was 65. We observed that age at operation (OR: 2.518, 95% CI: 1.236–5.131, *p* = 0.011), the log-transformed value of preoperative CA 19 − 9 (OR: 1.306, 95% CI: 1.056–1.616, *p* = 0.014), and preoperative tumor differentiation (poorly differentiated vs. well-differentiated, OR: 4.785, 95% CI: 1.622–12.244, *p* = 0.013; poorly differentiated vs. moderately differentiated, OR: 2.416, 95% CI: 1.165–5.606, *p* = 0.017) significantly increased the potential of LN metastasis. Therefore, we constructed a nomogram using these variables to predict the potential of LN metastasis in patients with T1 AoV cancer (Fig. 1). By adding the points from each predictor, the total points and possibility of LN metastasis or regional LN recurrence are obtained. The AUC of the newly developed nomogram was 0.741.

**Table 4:** Binary logistic regression analysis for potential of lymph node metastasis using preoperatively measurable factors (*n* = 53)

Variables	Univariable *p*	OR	95% CI	Multivariable *p*
Age at operation > 65 years	0.031	2.518	1.236–5.131	0.011
Sex, male (ref. female)	0.947			
BMI	0.781			
Preop. jaundice	0.169			
Preop. CA 19 − 9 (Log)	0.003	1.306	1.056–1.616	0.014
Preop. NLR	0.393			
Preop. PLR	0.454			
Preop. serum amylase	0.045			
Preop. serum lipase	0.400			
Preop. tumor size	0.653			
Preop. tumor differentiation	0.026			
Poorly differentiated (ref. well)		4.785	1.622–12.244	0.013
Poorly differentiated (ref. moderately)		2.416	1.165–5.606	0.017

*OR* odds ratio, *CI* confidence interval, *BMI* body mass index, *Preop.* preoperative, *NLR* neutrophil-to-lymphocyte ratio, *PLR* platelet-to-lymphocyte ratio

## Discussion

Studies have reported the increasing incidence of ampullary carcinoma as well as its precursor lesions, i.e., ampullary adenomas [13, 14]. PD is the surgical standard for ampullary carcinomas; however, owing to its high morbidity, less invasive approaches such as endoscopic resection or surgical ampullectomy have been proposed as alternatives for patients with high-operative risk [9, 15, 16]. In our previous multicenter study including 486 patients with AoV cancer, the survival outcome between the TDA and PPPD groups did not significantly differ in patients with T1 disease [16]. Nevertheless, the improper removal of regional LNs is a major concern related to these non-radical local resections. This may result in poorer oncological outcomes compared with patients undergoing PD, particularly in those with the potential of LN metastasis [15]. In previous studies, 10∼45% of patients with T1 AoV cancer had LN metastasis; this was a significant predictor of recurrence and survival [16, 17]. In the present study, among 342 patients with T1 cancer, 48 (14.0%) presented with LN metastasis. Because consensus on the appropriate conditions for local excision is lacking, we aimed to identify proper candidates for local excision by elucidating the preoperative predictors for LN metastasis.

Survival analysis confirmed that LN metastasis is the most powerful prognosticator for both recurrence and mortality in patients with T1 AoV cancer. In addition to the presence of metastatic LNs, recent studies have investigated the proportion of metastatic LNs to all harvested LNs, also called LN ratio (LNR). Many studies have suggested that the number of involved LNs and LNR exhibits prognostic significance for resected AoV cancer [18, 19]; therefore, the updated AJCC staging system now stratifies the N stage based on the number of metastatic LNs [10]. However, surgeons with different surgical extents and pathologists from various institutions may affect the total number of harvested LNs. There additional studies are warranted to elucidate how to use the LN indices in AoV cancer as both pre- and postoperative prognostic factors.

NLR is a factor associated with OS. Researchers have actively explored inflammatory markers, such as NLR, PLR, lymphocyte-to-monocyte ratio, and advanced lung cancer inflammation index in biliopancreatic and other gastrointestinal tumors [20–22]. These markers can be measured via blood tests which are routinely performed in patients undergoing surgery, which is a key strength. However, whether the increase in inflammatory marker levels reflects chronic inflammation, an important carcinogenic pathway [23], or if it is caused by microenvironment of malignant cells [24] or tumor-induced biliopancreatic passage disturbance remains unknown. Moreover, data regarding the effect of anti-inflammatory treatment in patients with malignancy are scarce. Several studies have reported that anti-inflammatory drugs may have antitumor activity and decrease the incidence of some malignancies [25]. If anti-inflammatory treatment can change the carcinogenesis or tumor progression of ampullary cancer, the above-mentioned factors will emerge as important and potentially modifiable prognosticators for patients with AoV cancer.

In the present study, tumor differentiation was identified as an independent factor associated with RFS as well as a predictor for LN metastasis in T1 AoV cancer. Poorly differentiated tumors indicate a worse prognosis in many other malignancies [26]. In some solid tumors, tumor differentiation is a major consideration when deciding the treatment modality. For example, in early-stage gastric cancer, endoscopic resection should be carefully performed with stringent criteria in patients with poorly differentiated tumors [27]. Also, there was a study suggesting that the surgical extent (subtotal gastrectomy vs. total gastrectomy) should be different in individuals with poorly differentiated gastric cancer [28]. Considering that endoscopic examination and simultaneous endoscopic biopsy or endoscopic ampullectomy are often performed in patients with newly diagnosed AoV cancers, tumor differentiation can play a vital role in deciding the appropriate surgical extent for patients with resectable AoV cancer. In this regard, our institution is planning additional research to develop a prediction model using preoperative pathological biopsy slides to select appropriate extent of resection and estimate the prognosis of patients with AoV cancer preoperatively.

In our logistic regression model for LN metastasis, older age (> 65 years) increased the risks of LN metastasis in T1 AoV cancer. This finding is inconsistent with that of previous studies, which have mostly reported an inverse relationship between age and the probability of LN metastasis. Researchers have identified the relationship between young age and higher odds of LN metastasis in breast, endometrial, and colon cancers [29–31]. On the other hand, in patients with Hurthle cell carcinoma, a rare thyroid tumor, older patients are more likely to exhibit LN metastasis [32]. Because patient age should be considered when deciding treatment plans such as surgical extent or adjuvant therapy, further study is necessary to investigate oncological implications of age and the association between age and LN status in AoV cancer.

CA 19 − 9 is a well-known prognostic factor in various hepatobiliary malignancies. Several studies reported that CA 19 − 9 was related to survival of patients undergoing surgical resection for AoV cancer [33]. This association can be explained by the production mechanism of the protein. CA 19 − 9 releases from ductal cells of biliopancreatic system, and the level of CA 19 − 9 increases when inflammation or obstruction presents in biliary tract or pancreatic duct [34]. Tumors arising from AoV cause obstruction of bile duct or pancreatic duct, so it is plausible that patients with AoV cancer present with elevated level of serum CA 19 − 9. Its significance in predicting prognosis after treatment of biliopancreatic malignancies including AoV cancer has been widely reported [33–35]. In the present study, elevated CA 19 − 9 level was also an independent factor predicting LN metastasis. A major limitation is its clinical utility as a tumor marker for screening and diagnosis [36, 37]. There is need of further investigation on potential utilization of the marker and its optimal cut-off values for AoV cancer.

The present study has several limitations. First, its multicenter retrospective design is a major limitation. Heterogeneity exists regarding preoperative evaluation, patient selection, decision on surgical extent, and postoperative follow-up. Particularly, there is a lack of details regarding postoperative adjuvant treatment. Since the role of adjuvant chemotherapy or chemoradiotherapy in resected AoV cancer remains unclear, there was no established policy regarding adjuvant treatment in both institutions and no consensus has been made among oncologists involved in the study. Further research should be performed to elucidate the role of adjuvant treatment and the potential of neoadjuvant chemotherapy in AoV cancer, so that the predictive nomogram might also be used to select proper candidates for pre- or postoperative chemotherapy. Second, the study is vulnerable to selection bias because only patients who underwent surgical ampullectomy were included, and the data of patients who underwent endoscopic ampullectomy for early AoV cancer were not included. Also, the proportion of patients with TDA to those with PD is very small, and more than a half of the TDA group did not have data on nodal status, which was the important outcome of the present study. Third, preoperative variables, data on nodal involvement such as the presence of enlarged LNs from preoperative image scans were not included. Above all, the standard for ampullary cancer is still PD, until any other researches provide strong evidence regarding oncologic safety of TDA. By the result of the present study, we might obtain information on the possibility of LN metastasis preoperatively, not the definitive surgical plan for patients. We would be able to select patients who need LN dissection, but the evidence is weak. Despite these limitations, we collected information on a great number of patients with early AoV cancer and proposed a simple nomogram using three factors for predicting LN metastasis preoperatively. By determining the possibility of LN metastasis preoperatively, physicians would assess the surgical risk and oncologic necessity for radical surgery including LN dissection and present patients with the best possible option. In future studies, we aimed to combine clinical variables with data from preoperative images and biopsies to improve the predictive power of the nomogram.

## Data Availability

The datasets used and/or analysed during the current study available from the corresponding author on reasonable request.

## Abbreviations

AoV: Ampulla of Vater
LN: Lymph node
CA 19 − 9: Carbohydrate antigen 19 − 9
PD: Pancreaticoduodenectomy
TDA: Transduodenal ampullectomy
NLR: Neutrophil-to-lymphocyte ratio
PLR: Platelet-to-lymphocyte ratio
AJCC: American Joint Committee on Cancer
RFS: Recurrence-free survival
HR: Hazard ratio
CI: Confidence interval
OR: Odds ratio
AUC: Area under the curve
ASA: American Society of Anesthesiology
